# A circulating microRNA panel as a novel dynamic monitor for oral squamous cell carcinoma

**DOI:** 10.1038/s41598-023-28550-y

**Published:** 2023-02-03

**Authors:** Yudan Piao, Seung-Nam Jung, Mi Ae Lim, Chan Oh, Yan Li Jin, Hae Jong Kim, Quoc Khanh Nguyen, Jae Won Chang, Ho-Ryun Won, Bon Seok Koo

**Affiliations:** 1 Department of Medical Science, Chungnam National University College of Medicine, Daejeon, Republic of Korea; 2 Department of Otolaryngology-Head and Neck Surgery, Chungnam National University College of Medicine, Daejeon, 35015 Republic of Korea; 3 Department of Otolaryngology-Head and Neck Surgery, Chungnam National University College of Medicine, Chungnam National University Sejong Hospital, 20, Bodeum 7-ro, Sejong, 30099 Republic of Korea

**Keywords:** Cancer, Computational biology and bioinformatics

## Abstract

Oral squamous cell carcinoma (OSCC) has high recurrence and mortality rates despite advances in diagnosis and treatment. Therefore, it is necessary to identify new biomarkers for early detection, efficient monitoring, and prognosis prediction. Since microRNA (miRNA) is stable and detectable in serum, it has been reported to inform the diagnosis and monitor disease progression through liquid biopsy. In this study, a circulating specific miRNA panel in OSCC patients was developed, and its usefulness as a dynamic monitor was validated. Small RNAs were extracted from the serum of OSCC patients (n = 4) and normal controls (n = 6) and profiled using next-generation sequencing. NGS identified 42 differentially expressed miRNAs (DEmiRNAs) in serum between patients with OSCC and healthy controls, with threefold differences *(p* < 0.05). Combining the 42 DEmiRNAs and The Cancer Genome Atlas (TCGA) databases OSCC cohort, 9 overlapping DEmiRNAs were screened out. Finally, 4 significantly up-regulated miRNAs (miR-92a-3p, miR-92b-3p, miR-320c and miR-629-5p) were identified from OSCC patients via validation in the Chungnam National University Hospital cohort. Application of the specific miRNA panel for distinguishing OSCC patients from healthy controls produced specificity and sensitivity of 97.8 and 74%, respectively. In addition, the serum levels of these 4 miRNAs significantly decreased after complete surgical resection and increased after recurrence. We suggest that circulating 4-miRNA panel might be promising non-invasive predictors for diagnosing and monitoring the progression of patients with OSCC.

## Introduction

Oral squamous cell carcinoma (OSCC) is the most common type of oral cancer, accounting for approximately 350,000–400,000 cases per year. OSCC is twice as common in male than female due to risk factors, such as tobacco, alcohol and HPV. According to statistics, OSCC is the 6th and 8th particularly for incidence and mortality in both men, respectively^1^. Due to the high occurrence of secondary carcinoma and tumor heterogeneity, OSCC is often diagnosed in an advanced state with a poor prognosis^2^. Even though most cases of OSCC could be managed with complete surgical resection alone or a combination of ionizing radiation or chemo-radiation therapy, a certain proportion of advanced OSCCs remain unresponsive to treatment or exhibit loco-regional recurrence, resulting in a mortality rate of 50%^3,4^.

In the diagnosis and prevention of OSCC, emphasis is placed on identifying potential malignant lesions of the oral mucosa and local diseases that promote chronic inflammation, mainly relying on objective clinical examinations and surgical biopsy^5,6^. Although surgical biopsy is the gold standard for the diagnosis of OSCC, it is somewhat invasive and can sometimes be harmful to patients^7^. Moreover, conventional biopsy is temporally and spatially limited and often provides a brief snapshot of a single region of a heterogeneous tumor^8^. Therefore, it is crucial to find promising non-invasive biomarkers for monitoring or patient surveillance and further illuminate the pathogenesis of OSCC regarding tumor behavior at the molecular level. Blood samples are relatively easy to collect in a minimally invasive manner, and increasingly many recent studies have suggested that circulating microRNAs (miRNAs) are promising as potential biomarkers for disease diagnosis and monitoring^9,10^.

miRNAs are small, non-coding RNAs of 18–25 nucleotides in length that have been linked to essentially all known pathological and physiological processes, including cancer. Recent studies have reported that miRNAs can not only be utilized for diagnosis and prognosis, but also play integral and convoluted roles in the regulatory network of cancer. miRNA have been reported as diagnostic biomarkers for many cancers, including head and neck cancer^11,12^. However, the approach of using tissue-derived miRNA in surveillance or prognosis is commonly invasive in nature which may impede the screening. Furthermore, previous studies have demonstrated that miRNA can be quite stable in serum due to its protection from endogenous RNase activity and that it is readily detected by various assays^13,14^, presents the possibility to exploiting circulating miRNAs as biomarkers for early-stage cancer. Therefore, serum miRNA panel signatures have recently been identified as promising candidate biomarkers for liquid biopsy. However, studies have rarely examined circulating miRNA expression in patients with OSCC, leading to little noticeable and reliable signatures.

The aim of this study was to explore and validate the possibility that circulating miRNAs could overcome the limitations of tissue biopsy and act as potential biomarkers in liquid biopsy for the early diagnosis and dynamic monitoring of disease progression in OSCC patients.

## Materials and methods

### Patient and sample collection

Serum samples from 27 patients with OSCC and 21 age- and sex-matched healthy individuals were obtained at the Chungnam National University Hospital (CNUH) (Daejeon, Republic of Korea), between January 2017 and December 2019. The clinical information of patients with OSCC were summarized in Table S1. Tumor tissues and adjacent non-tumor tissues were collected from 7 patients with OSCC. All patients with OSCC were enrolled at the initial diagnosis, and the pathological diagnoses were subsequently confirmed. The study participant provided an informed consent form before participating. The Institutional Review Board of CNUH approved this study (CNUH 2019-07-041). All methods were performed in accordance with the Institutional Review Board of CNUH guideline and regulation.

### Next-generation sequencing and analysis

Serum samples from 4 OSCC patients and 6 age- and sex-matched healthy controls were selected from CNUH cohort for next-generation sequencing^10^. The clinical information of patients with OSCC for NGS were presented in Table S2. Whole-transcriptome next-generation sequencing was performed by Macrogen Inc. (Seoul, Republic of Korea). Briefly, extracted RNA samples were used to prepare small RNA libraries using SMARTer smRNA-Seq Kit protocol and sequenced using a HiSeq 2500 sequencer (Illumina, San Diego, CA, USA), following the HiSeq 2500 System User Guide Document #15035786 v02 HCS 2.2.70 protocol. After sequencing, the raw sequence reads were filtered based on quality determined by the phred quality score at each cycle (Table S3). Both the trimmed reads and non-adapter reads as processed reads were used, to do analyzing long target (≧ 50 bp).The processed reads were gathered forming a unique cluster. In order to eliminate the effect of large amounts of ribosomal RNA (rRNA) from this study, the read was aligned to the rRNA sequence. rRNA removed reads were sequentially aligned to reference genome (UCSC Homo sapiens reference genome (GRCh37/hg19)), miRBase v21 and non-coding RNA database, RNAcentral 10.0 to classify known miRNAs and other type of RNA such as tRNA, snRNA, snoRNA etc. Novel miRNA prediction was performed by miRDeep2. The read counts for each miRNA were extracted from mapped miRNAs, differentially expressed miRNAs (DEmiRNAs) were determined through comparing across conditions each miRNA using statistical methods. Detailed work flow of sequencing and analysis were additionally described in the supplementary material. Figure S1 represents the small RNA composition of each sample.

### Bioinformatics

Differentially expressed miRNAs (DEmiRNAs) between the evaluated groups were estimated using DESeq2 and edgeR^15^. The screening criteria were a fold change > 3 and *p* < 0.05. All genomic data of OSCC from The Cancer Genome Atlas (TCGA) were obtained from a specific portal (https://tcga-data.nci.nih.gov) and cancer browser (https://genome-cancer.ucsc.edu). To select miRNA differentially expressed between patients with OSCC and normal controls, false discovery rate-adjusted *p* values (< 0.05) were used to correct, using the Benjamin-Hochberg method. A volcano map, heatmap, and cluster analysis were conducted using an online analysis tool (https://www.chiplot.online/), a free online platform for data analysis and visualization. The target genes of miRNAs were predicted with the TargetScan 8.0 database (www.targetscan.org). Functional annotation was performed using the Database for Annotation, Visualization and Integrated Discovery (https://david.ncifcrf.gov/), a web-accessible tool for Gene Ontology (GO) and Kyoto Encyclopedia of Genes and Genomes (KEGG) pathway enrichment analysis. A network analysis of miRNA-mRNA interactions was carried out using Cytoscape (version 3.7.1), an open bioinformatics software.

### RNA extraction and quantitative real time polymerase chain reaction (qRT-PCR)

Circulating miRNA was isolated from 200 μL of serum for RNA purification using miRNeasy serum/plasma kits (Qiagen, Hilden, Germany) according to the manufacturer’s protocol. Total RNA was extracted from tissue samples using the TRIzol reagent (Invitrogen, Waltham, MA, USA). The SYBR Green qRT-PCR assay was used for miRNA quantification. Total miRNA was used as the template for cDNA synthesis with miScript II RT Kit (Qiagen, Hilden, Germany) according to the manufacturer’s instructions. For miRNA analysis, qRT-PCR was performed using the miScript SYBR Green PCR kit (Qiagen, Hilden, Germany) with the manufacturer-provided miScript assays, using the universal primer and miRNA-specific forward primers with the 7500 system. The miRNA-specific primers were obtained from the miScript primer assays, the miRCURY LNA miRNA PCR Assay (Qiagen, Hilden, Germany), and Bioneer (Daejeon, Korea). All primer sequences used for qRT-PCR are listed in Table S4. miR-16 and miR-423-5p were used as references for serum miRNA analysis, and U6 small nuclear (RNU6) was used as the reference for the tissue expression of miRNA. At the end of the PCR cycles, melting curve analyses were performed. Each sample was run in triplicate for analysis. The 2 − ΔΔCT method was used to analyze the expression levels of miRNA.

### Statistical analysis

All statistical analyses were performed using SPSS for Windows version 26 (IBM Corp., Armonk, NY, USA) and GraphPad Prism 8 (GraphPad Software, La Jolla, CA, USA). All experiments in the CNUH OSCC patient cohort were repeated three times. The data on the expression differences of miRNA between patients with OSCC and healthy controls, and between the serum samples and tissue samples from the same patients were analyzed using Mann–Whitney U test or independent *t*-test. The data on the expression differences of miRNA between the same patients before and after surgery were analyzed by paired *t*-test. Data are expressed as means ± SD. *p < 0.05, **p < 0.01, ***p < 0.001. Receiver operating characteristic (ROC) curves were used to analyze the diagnostic value of DEmiRNAs. A logistic regression model was constructed to determine the predicted probability of the combination of the 4 miRNAs. Pearson correlation coefficients were used to compare the miRNA levels in serum and tissue. The independent *t*-test was used to identify possible associations between miRNA concentrations and clinicopathological features of OSCC patients. The levels of miRNA in each group were presented as mean ± standard deviation (SD). All *p* values were two-sided, and a *p* value < 0.05 was considered statistically significant.

## Results

### Serum miRNA profiling to identify differential expression between healthy individuals and OSCC patients

**Figure 1:**
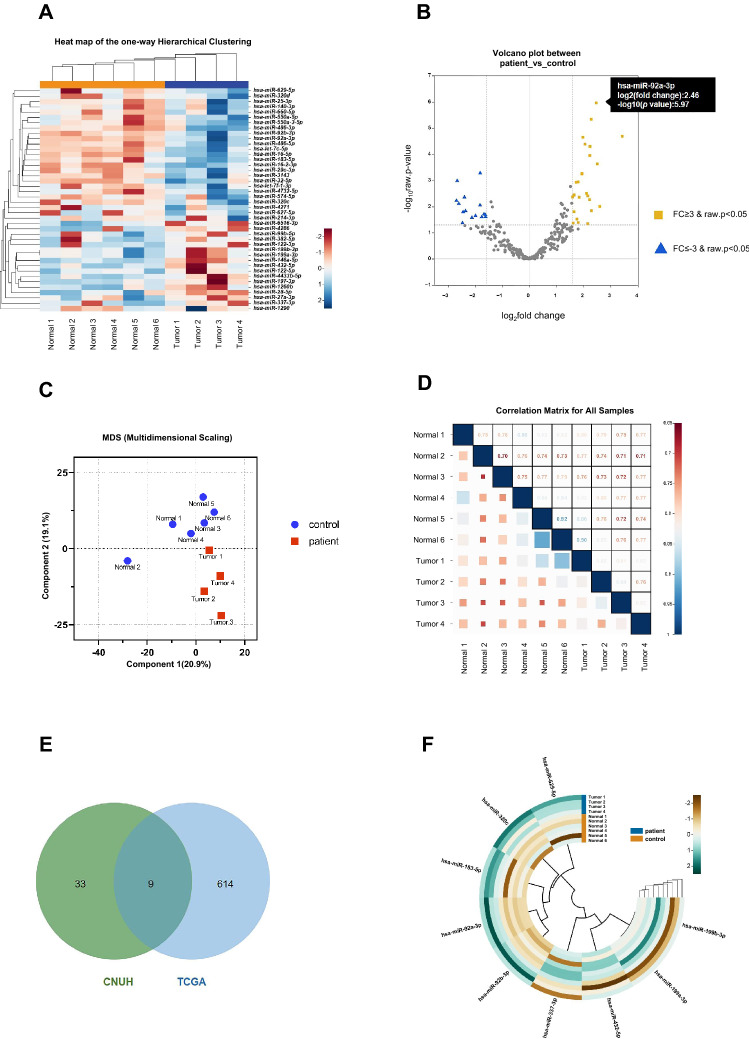
miRNA profiling identifies differential expression in OSCC. (**A**) Heatmap of miRNAs that were differentially expressed between oral squamous cell carcinoma (OSCC) patients and normal controls *(p* < 0.05). Four pre-treatment OSCC serum samples and 6 normal serum samples are shown in the heatmap. (**B**) A volcano plot shows that many miRNAs were significantly different between normal and OSCC patients. Yellow: up-regulation with a fold change of more than 3; blue: down-regulation with a fold change of more than −3 (*p* > 0.05). (**C**) Principal component analysis (PCA). The fold change in expression between matched normal and tumor samples was used to perform PCA. (**D**) Heatmap of correlations based on the OSCC and normal serum samples. The correlogram shows correlation coefficients for all pairs of variables with coefficients colored based on their sign. (**E**) Venn diagram of differentially expressed miRNAs (DEmiRNAs) obtained from The Cancer Genome Atlas (TCGA) database and RNA sequencing results (fold change ≥ 3 or ≤ −3, *p* < 0.05). The Venn diagram shows that there are 9 overlapping DEmiRNAs. (F) Heatmap of one-way hierarchical clustering revealed 9 DEmiRNAs. The heatmap, volcano plot and PCA plot were conducted using an online tool ChiPlot (https://www.chiplot.online/).

To identify potential circulating miRNA biomarkers of OSCC, we measured serum expression levels in 4 patients with OSCC and 6 healthy controls by NGS (small RNA-sequencing). The overview of the research workflow is illustrated in Fig. S2. In the initial screening, we identified 272 DEmiRNAs between patients with OSCC and healthy controls based on the exactTest using DESeq2 and edgeR. We screened out 42 DEmiRNAs, including 26 up- and 16 down-regulated DEmiRNAs, based on 2 criteria: (1) compared to the healthy group, the DEmiRNAs in the OSCC group had at least a threefold change in expression; and (2) the *p* values had statistical significance (*p* ≤ 0.05) with adjustment by the Benjamin-Hochberg procedure for multiple testing correction (Fig. 1A). The volcano plot directly presents the miRNA expression levels, and the most significantly up-regulated miRNA was shown to be miR-92a-3p, which had a log_2_ fold change of 2.46 (Fig. 1B). Using each sample’s normalized value, principal component analysis showed a circulating miRNA expression signature that segregated the serum samples of OSCC from those of healthy controls (Fig. 1C). We also identified the similarity between samples of the same group through Pearson correlation coefficients for the normalized values (Fig. 1D). Studies of microRNA in serum specific to cancer patients is based on the premise that it is expressed through the process of being released into the bloodstream from cancer tissue. The mechanism is not yet clear, but it is known to be a product of tumor cell death and dissolution or release from tumor-derived microsomes or exosomes^16,17^. Therefore, to discover specific candidate miRNAs, we combined our small RNA-sequencing results and data from TCGA, a large-scale tissue-derived database. Finally, 9 miRNAs were identified as candidates due to their differential expression in both the serum and tissue of OSCC patients. A Venn diagram (Fig. 1E) shows the screening pattern, and Fig. 1F and Table S5 present the 9 candidates, including 5 up- and 4 down-regulated DEmiRNAs. To further investigate the functions and pathways by which the dysregulation of the DEmiRNAs influences OSCC development, we predicted the target genes of the 9 DEmiRNAs and performed GO and KEGG pathway enrichment analysis. The target genes of the 9 DEmiRNAs participated in cancer progression-related processes, such as the PI3K-Akt signaling pathway and signaling pathways regulating choline metabolism (Fig. S3A-B). For biological processes, cellular components, and molecular function, the target genes of DEmiRNAs were significantly concentrated in cellular nitrogen compound metabolic process, organelle, ion binding, and biosynthetic process (Fig. S3C-D). Next, we identified downstream targets associated with DEmiRNA that could play a regulatory role in OSCC progression. The miRNA target predictions were performed using the TargetScan databases, and then we used Cytoscape software to visualize and analyze the predicted data for interactions in miRNA-mRNA regulatory networks (Fig. S3E-F). It was confirmed that one miRNA regulated the signal transduction pathway in association with several mRNAs and was also interconnected with other miRNAs.

### Validation of the candidate miRNAs in the CNUH OSCC patient cohort by qRT-PCR

To search for and validate potential miRNA signatures to distinguish OSCC patients from healthy controls, we planned to validate the 9 candidate DEmiRNAs in the CNUH OSCC patient cohort. We collected serum samples from 23 OSCC patients with different subsites (tongue, n = 18; buccal mucosa, n = 2; retromolar trigone, n = 2; and floor of mouth mucosa, n = 1) and 15 healthy controls from the cohort for validation by qRT-PCR. The clinical information of patients with OSCC were summarized in Table S1.

**Figure 2:**
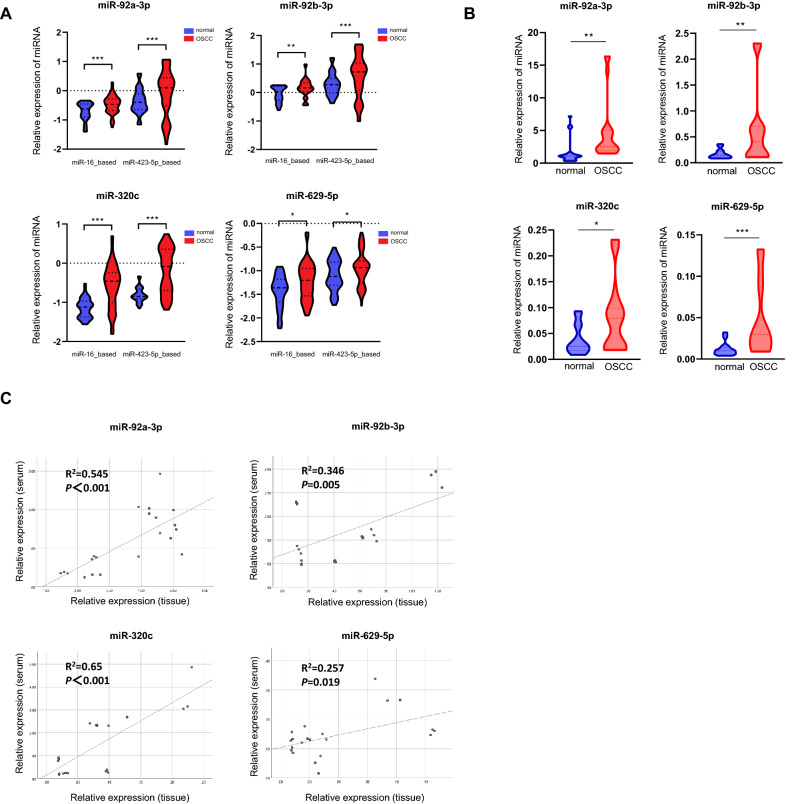
qRT-PCR validation data of selected differentially expressed miRNAs. (**A**) Serum samples. The expression levels of miRNAs were calculated using the 2^-ΔΔCT^ method, and the results are presented as relative log_10_ fold changes. (**B**) Tissue samples. Three biological duplicates were included for each condition. Significantly deregulated miRNA expression was found in patients with oral squamous cell carcinoma (OSCC) versus the healthy controls. (**C**) Scatter plots showing the relative expression of 4 miRNAs in OSCC serum and tissue samples (Pearson correlation).

Previous studies have shown that RNU6 can generally be used as an endogenous control (EC) to normalize the expression of miRNA in tissue or cells, but it is unstably expressed in the plasma and serum^18^. miR-16 is more stable in different populations’ serum and also relatively stable after freezing and thawing^19–21^. Based on a proposed protocol for circulating miRNA normalization^22^, we chose to combine miR-16 and miR-423-5p^20^ as the EC to improve the accuracy of quantification. When miR-16 and miR-423-5p were used as the reference control to analyze the qRT-PCR results, we found that 4 miRNAs (miR-92a-3p, miR-92b-3p, miR-320c and miR-629-5p) from the 9 candidate DEmiRNAs were present at significantly higher levels in the OSCC group than in healthy controls (Fig. 2A). The remaining 5 miRNAs did not show significant results in the validation process (Fig. S4). To verify whether serum miRNA expression levels were associated with those in tissue, we selected 7 pairs of OSCC tissues (matched tumor and neighboring normal tissues) from the CNUH cohort to analyze the expression profiles of the 4 candidate miRNAs. The qRT-PCR results indicated that the expression levels of miR-92a-3p, miR-92b-3p, miR-320c and miR-629-5p were also significantly higher in the OSCC tissues than in the normal controls (2.79-, 3.79-, 2.23- and 3.97-fold, respectively, *p* < 0.05) (Fig. 2B). Next, we calculated Pearson correlation coefficients using the qRT-PCR results of serum and tissue from the CNUH cohort. A prominent positive correlation was found between the serum and tissue expression of the specific circulating miRNA panel (Fig. 2C). These results show that the specific circulating miRNA panel, including miR-92a-3p, miR-92b-3p, miR-320c, and miR-629-5p, may serve as a reliable set of biomarkers for the detection of OSCC.

### Predictive value and clinicopathological correlations of the specific circulating miRNA panel for OSCC

**Figure 3:**
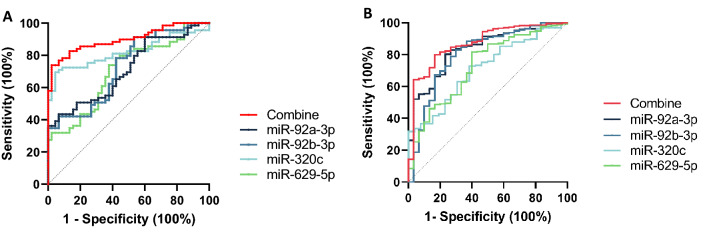
Diagnostic value of the expression of 4 candidate miRNAs. Receiver operating characteristic curve analyses of the specific 4-miRNA panel in (**A**) the Chungnam National University Hospital (CNUH) cohort and (**B**) the The Cancer Genome Atlas (TCGA) cohort.

To estimate the potential diagnostic threshold of the expression of 4 candidate miRNAs in serum for OSCC, we conducted a ROC curve analysis for discriminating between patients with OSCC and healthy controls. The ROC curve is the most popular graphical tool for evaluating the diagnostic power of a biomarker. It provides a comprehensive overview of trends for sensitivity over all cut-offs, and thus provides information about the relationship between the specificity and sensitivity of a biomarker^23^. A comparison of the OSCC group and the healthy control group indicated that miR-92a-3p had an area under the curve (AUC) of 0.7108 [95% confidence interval (CI) = 0.6174–0.8042], with specificity and sensitivity values of 0.9333 and 0.4348, respectively; miR-92b-3p had an AUC of 0.7269 (95% CI = 0.6333–0.8204), with specificity and sensitivity values of 0.4667 and 0.913, respectively; miR-320c had an AUC of 0.8206 (95% CI = 0.7432–0.898), with specificity and sensitivity values of 0.9556 and 0.6957, respectively; and miR-629-5p had an AUC of 0.7011 (95% CI = 0.6045–0.7977), with specificity and sensitivity values of 0.6222 and 0.7391, respectively (Fig. 3A). When combining the 4 miRNAs for the analysis, the AUC was 0.899 (95%CI = 0.8431–0.9547) and the specificity and sensitivity values were 0.978 and 0.739, respectively; these values were higher than in the individual analyses (Fig. 3A). We subsequently verified the ROC analysis results using the TCGA cohort. As shown in Fig. 3B, the combination of 4 miRNAs yielded an AUC of 0.8698, with a specificity of 0.8333 and a sensitivity of 0.7993 for OSCC diagnosis. Individually, for miR-92a-3p, miR-92b-3p, miR-320c, and miR-629-5p, the AUC was 0.8342, 0.8025, 0.7097, and 0.7273, respectively (Fig. 3B).

Next, we used the clinical information from the TCGA database to investigate whether the elevated levels of the 4 miRNAs in OSCC patients were associated with clinicopathological characteristics. The distribution of clinicopathological factors and the expression levels of 4 candidate miRNAs are presented in Table 1. Higher histological grades were associated with increased expression of all 4 miRNAs. In particular, elevated miR-92a-3p and miR-92b-3p levels were significantly associated with lymph node metastasis. In addition, the expression of miR-92b-3p was significantly elevated in patients with lymphovascular invasion (Table 1).

**Table 1:** Correlations between clinicopathological characteristics and expression levels of miRNA in the TCGA dataset.

Characteristics	Number	miR-92a-3p	*p*	miR-92b-3p	*p*	miR-320c	*p*	miR-629-5p	*p*
Age									
< 60	150	13.521 ± 0.686	0.287	6.418 ± 0.951	0.169	0.884 ± 0.445	0.677	7.033 ± 0.645	0.518
≥ 60	114	14.024 ± 0.557		7.047 ± 0.726		0.876 ± 0.497		7.714 ± 0.600	
Clinical N stage									
N0-N1	193	13.472 ± 0.799^a^	**0.002**	6.293 ± 0.902^a^	**0.000**	0.867 ± 0.454	0.145	7.043 ± 0.633	0.902
N2-N3	71	13.710 ± 0.738^a^		6.705 ± 0.993^a^		0.931 ± 0.426		7.036 ± 0.691	
Clinical T stage									
T1-T2	102	13.533 ± 0.773	0.697	6.427 ± 0.979	0.919	0.935 ± 0.495	0.098	7.010 ± 0.646	0.382
T3-T4	162	13.562 ± 0.792		6.419 ± 0.929		0.865 ± 0.414		7.078 ± 0.648	
Overall clinical stage									
I + II	76	13.451 ± 0.713	0.101	6.352 ± 0.908	0.345	0.894 ± 0.490	0.945	7.059 ± 0.577	0.705
III + IV	188	13.584 ± 0.802		6.445 ± 0.962		0.891 ± 0.432		7.033 ± 0.668	
Lymphovascular invasion present									
Yes	91	13.559 ± 0.852	0.534	6.487 ± 0.907^a^	**0.012**	0.930 ± 0.425	0.078	6.946 ± 0.636	0.077
No	173	13.613 ± 0.719		6.244 ± 0.830^a^		0.840 ± 0.452		7.069 ± 0.602	
Neoplasm histologic grade									
I + II	208	13.512 ± 0.746^a^	**0.026**	6.305 ± 0.906^a^	**0.000**	0.851 ± 0.431^a^	**0.005**	7.019 ± 0.644^a^	**0.040**
III + IV	56	13.689 ± 0.880^a^		6.690 ± 0.961^a^		0.980 ± 0.465^a^		7.048 ± 0.643^a^	
Pathologic N stage									
N0-N1	169	13.523 ± 0.732	0.551	6.242 ± 0.949^a^	0.001	0.891 ± 0.440	0.420	7.061 ± 0.598	0.638
N2-N3	95	13.570 ± 0.842		6.551 ± 0.922^a^		0.854 ± 0.436		6.928 ± 0.682	
Pathologic T stage									
T1-T2	110	13.489 ± 0.821	0.487	6.361 ± 0.976	0.634	0.881 ± 0.473	0.745	6.999 ± 0.610	0.146
T3-T4	154	13.542 ± 0.763		6.405 ± 0.915		0.866 ± 0.423		7.078 ± 0.649	
Overall pathologic stage									
I + II	70	13.415 ± 0.786	0.121	6.415 ± 0.968	0.760	0.831 ± 0.465	0.327	7.101 ± 0.609	0.141
III + IV	194	13.549 ± 0.783		6.384 ± 0.926		0.881 ± 0.437		6.999 ± 0.644	

Significant values are denoted by bold font.

*TCGA* The Cancer Genome Atlas, *T* tumor size, *N* lymph nodes, *p* *p* value.

*p* < 0.05, *p* values calculated by the independent *t*-test.

### Application of the circulating miRNA panel for dynamic monitoring of OSCC

**Figure 4:**
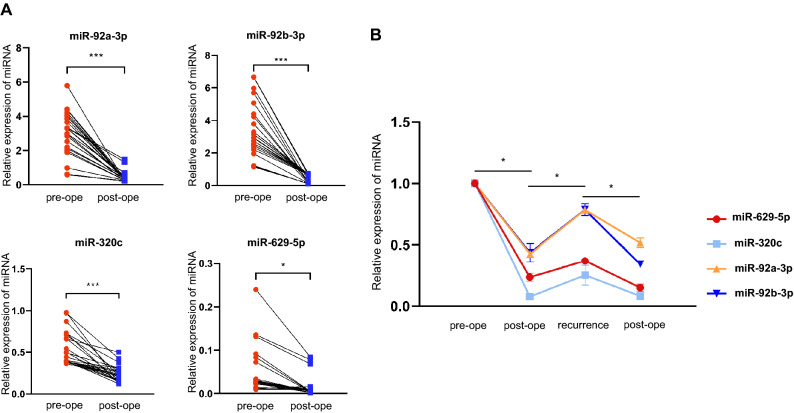
Circulating levels of the 4 miRNAs contribute to the monitoring of tumor treatment responses. (**A**) Serum expression of candidate miRNAs in patients with oral squamous cell carcinoma (OSCC) (n = 8) before and after surgical removal of the primary tumor. (**B**) Four miRNAs follow the process in a patient with OSCC recurrence. All *p *values were determined by the paired *t*-test.

Subsequently, we selected 8 patients with OSCC [tongue, n = 6; buccal mucosa, n = 1; retromolar trigone, n = 1) who were regularly followed up and collected preoperative and postoperative (3 months after surgery on average) serum samples for validation by qRT-PCR, to explore whether curative treatment of the tumor would affect the expression levels of circulating miRNA in the serum. The serum expression levels of miR-92a-3p, miR-92b-3p, miR-320c and miR-629-5p remarkably decreased after complete surgical resection (Fig. 4A). One of the patients experienced recurrence 9 months after initial complete resection, and the expression levels of the 4 miRNAs in the serum increased again, followed by a decrease after surgical resection at the recurrent site (Fig. 4B). The clinical course of recurrent patient is illustrated in Fig. S5. Therefore, our data demonstrate the possibility of using this panel with 4 circulating miRNAs for dynamic monitoring to evaluate the treatment results and recurrence of OSCC.

## Discussion

In this study, we performed initial screening using NGS to measure the expression levels of serum miRNAs in patients with OSCC and healthy controls. We then compared the selected representative DEmiRNAs with data from TCGA, a large-scale tissue-derived database, to obtain 9 overlapping candidate miRNAs. After validation of the candidate miRNAs in the CNUH cohort via qRT-PCR, we explored the expression levels of 4 miRNAs (miR-92a-3p, miR-92b-3p, miR-320c and miR-629-5p), which were significantly increased in both serum and tissue samples of patients with OSCC compared with a healthy cohort. Furthermore, we identified that circulating 4 miRNAs were potential non-invasive biomarkers to support the dynamic monitoring of OSCC. In a ROC curve analysis to evaluate the diagnostic value of these 4 candidate miRNAs, we found that their combination could achieve better diagnostic accuracy than each miRNA individually, with an AUC of 0.899, sensitivity of 74%, and specificity of 97.8%. Consequently, our study suggests that this specific circulating miRNA panel, including miR-92a-3p, miR-92b-3p, miR-320c and miR-629-5p, could be a powerful potential set of molecular biomarkers for discriminating between individuals with OSCC and healthy individuals.

Among the 4 candidate miRNAs, miR-92a-3p has been reported as a biomarker associated with head and neck cancer^24^. Beyond OSCC, it has been reported that miR-92a-3p also plays a role as an oncogenic component in gastric cancer^25^, esophageal squamous cell cancer^26^, cervical cancer^27^, and breast cancer^28^. Hu et al. also found that the expression of miR-92a-3p was increased both in colorectal cancer cells and in patients’ serum by secreting exosomes; furthermore, its expression was closely linked with metastasis and chemotherapy resistance in colorectal cancer patients^29^. miR-92b-3p has been reported as a suppressor due to down-regulation in several cancers^30^. However, recent studies have also proposed that miR-92b-3p plays an oncogenic role for proliferation, migration, and invasion of a few cancers, such as glioblastoma^31^, colorectal cancer^32^, renal cell carcinoma^33^, and gastric cancer^34^. Exosomal miR-92b-3p has also been considered as a potential dynamic biomarker to monitor chemoresistance in small-cell lung cancer^35^.and synovial sarcoma^36^. miR-320c exerts an inhibitory effect on several cancers^37^; however, it has recently been found that the expression levels of miR-320c in plasma of nasopharyngeal carcinoma patients were higher than in the control group^38^. qRT-PCR revealed that miR-320c was significantly up-regulated in plasma exosomes of patients with colon cancer^39^. In esophageal squamous cell carcinoma, miR-320c, miR-92a-3p, and 2 other miRNAs were identified as potential biomarkers because of their higher expression level in the serum^40^. Previous studies have confirmed the diagnostic value of miR-629-5p levels as an oncogenic miRNA in patients with prostate cancer^41^, hepatocellular carcinoma^42^, and lung adenocarcinoma^43^. As noted above, there are several existing studies on the 4 miRNAs that we identified. However, our study is meaningful in that it is the first study to directly confirm the expression of these miRNAs in the serum of OSCC patients through NGS, and the credibility of the findings was enhanced by conducting a combined analysis with the TCGA database.

The fundamental mechanism by which circulating miRNAs are released from tissue into body fluids is unclear. Based on previous research, the possible processes for the transference of circulating miRNAs between secretory and recipient cells are as follows: (1) passive leakage of necrotic or injury cells; (2) active secretion via extracellular vesicles, RNA binding proteins, or lipoproteins; and (3) direct transfer through cell gap junction-mediated intercellular communication^44^. Our qRT-PCR validation results confirmed that the expression levels of the 4 miRNAs in the specific circulating miRNA panel were elevated in both serum and tissues in the same patients, compared with healthy controls. Furthermore, independent positive associations were also found between the circulating miRNAs and OSCC tissues. We indirectly demonstrated the relationship between serum and tissue miRNA expression through these data. This association has also been proven in previous research. Xu et al. identified that the expression level of circulating miR-1290 was positively associated with tumor size, tumor differentiation, and the TNM stage in patients with gastrointestinal tumors. After treating a xenograft model with 5-fluorouracil, the expression level of circulating miR-1290 remarkably decreased^45^.

Of particular note, in the present study, serum samples were collected before and after surgery in 8 OSCC patients, and it was confirmed that the serum levels of the specific 4-miRNA panel significantly decreased after complete surgical resection in all patients. Additionally, although data on recurrence were available from only 1 patient, the serum expression levels of the 4 miRNAs increased again during recurrence, followed by a decrease after surgical resection. Therefore, this study is meaningful in that it directly confirmed that these miRNAs could be used as biomarkers for monitoring treatment outcomes and tumor recurrence, as well as to predict the prognosis.

Although previous studies have provided abundant evidence for the potential clinical relevance of circulating miRNAs as cancer biomarkers in different types of malignancies, the accuracy of circulating miRNAs remains a matter of debate. Major issues include the sample choice, sample contamination, and the lack of a uniform EC for normalization^46^. In the last few years, saliva, plasma, and serum miRNA levels have been reported as biomarkers in OSCC. Environmental factors, such as dietary restrictions and differences in dental hygiene, affect the stability of miRNA in saliva^47^. Blood cell contamination is the main issue that impacts the accuracy of plasma/serum miRNA quantification^48^. Wang et al. reported that serum contained higher levels of specific miRNAs than did plasma^49^. Therefore, serum was selected as the optimal sample for measuring circulating miRNAs related to cancer in this study. In addition, to increase the reliability of circulating miRNAs expressed in the serum, two housekeeping miRNAs (miR-16 and miR-423-5p) were used for double-checking.

The main limitation of this study is the small size of the sample in which NGS and validation were performed. Therefore, further research is necessary with a larger cohort to assess the specific signature of this panel of 4 miRNAs. In addition, in-depth research on how each miRNA acts as an oncogenic driver, focusing on their mechanisms, should be conducted in the future.

In conclusion, our results suggested that the circulating specific miRNA panel, including miR-92a-3p, miR-92b-3p, miR-320c, and miR-629-5p, may serve as a promising set of non-invasive biomarkers for early diagnosis and monitoring of the prognosis of OSCC.

## Supplementary Information

Supplementary Information.

## Data Availability

The TCGA data presented in this study are openly available in a specific portal (https://tcga-data.nci.nih.gov) and cancer browser (https://genome-cancer.ucsc.edu). Further information is available from the corresponding author upon request.

## Abbreviations

AUC: Area under the curve
BP: Biological processes
CC: Cellular components
CI: Confidence interval
CNUH: Chungnam National University Hospital
DEmiRNAs: Differentially expressed miRNAs
EC: Endogenous control
GO: Gene ontology
HNSCC: Head and neck cancer
KEGG: Kyoto encyclopedia of genes and genomes
MF: Molecular function
miRNA: MicroRNA
NGS: Next-generation sequencing
OSCC: Oral squamous cell carcinoma
PCA: Principal component analysis
ROC: Receiver operating characteristics
RT-PCR: Real-time polymerase chain reaction
TCGA: The cancer genome atlas
