# Supplementary material for: A circulating microRNA panel as a novel dynamic monitor for oral squamous cell carcinoma
Source: Sci Rep. 2023 Feb 3;13:2000. doi: 10.1038/s41598-023-28550-y (PMC9898506; doi:10.1038/s41598-023-28550-y)
Supplement: Supplementary file 1 — Supplementary Information. [file 41598_2023_28550_MOESM1_ESM.docx]

**Supplementary Materials for**

**A Circulating MicroRNA Panel as a Novel Dynamic Monitor for Oral Squamous Cell Carcinoma**

Yudan Piao^1^, Seung-Nam Jung^2^, Mi Ae Lim^2^, Chan Oh^1^, Yan Li Jin^1^, Hae Jong Kim^1^, Nguyen Quoc Khanh^1^, Jae Won Chang^1,2^, Ho-Ryun Won^1,2*^, Bon Seok Koo^1,2*^

*^1^Department of Medical Science, Chungnam National University College of Medicine, Daejeon, Republic of Korea*

^2^*Department of Otolaryngology-Head and Neck Surgery, Chungnam National University College of Medicine, Daejeon, Republic of Korea*

***** These authors contributed equally to this work as corresponding authors.

**Correspondence Authors:**

Bon Seok Koo MD, PhD.

Professor, Department of Otolaryngology-Head and Neck Surgery,

Chungnam National University College of Medicine,

266, Munhwa-ro, Jung-gu, Daejeon, 35015, Republic of Korea

Tel: +82-42-280-7690 Fax: +82-42-253-4059

E-mail: bskoo515@cnuh.co.kr

Ho-Ryun Won MD, PhD.

Professor, Department of Otolaryngology-Head and Neck Surgery,

Chungnam National University College of Medicine,

Chungnam National University Sejong Hospital

20, Bodeum 7-ro, Sejong, 30099, Republic of Korea

Tel: +82-44-995-4756 Fax: +82-44-995-5099

E-mail: hryun83@cnuh.co.kr

**Supplementary for Methods**

*Next-Generation Sequencing and Analysis*

Next generation sequencing (NGS) with Illumina (Hiseq2500) technology was used for the miRNA study. All the extracted RNA samples were used to prepare small RNA libraries using SMARTer smRNA-Seq Kit protocol. The raw reads were quality checked with FastQC. FastQC tool analyzes the base quality score distribution, sequence quality score distribution, average base content per read. The quality of produced data is determined by the phred quality score. The average quality at each cycle is created with FastQC. Phred quality score 20 means of 99% accuracy and reads over score of 20 are accepted as good quality. Unwanted sequences such as adapter sequences, primers, poly-A tails and other types of sequences are removed using Cutadapt 1.16 tool. For each sample, final processed reads are sequentially aligned to reference genome, miRBase v21 and non-coding RNA database (RNAcentral release 10.0) to classify known miRNAs and other types of RNA such as piRNA, tRNA, snRNA, snoRNA etc. To identify both known and novel miRNAs, the quality-controlled reads were aligned to the reference human genome (hg19). Genome mapping is processed by Bowtie and STAR using RSEM. Bowtie was subsequently used for miRDeep2 analysis using a genomic sequence. Known/novel miRNA predicted by miRDeep2 and other smRNAs matching RNAcentral were aligned using Bowtie and Bowtie2. HISAT2 and STAR was used to identify sequences that did spliced RNA, and align the transcript sequence. In order to reduce systematic bias, estimates the size factors from the count data and applies Trimmed Mean of M-values (TMM) normalization with edgeR R library. Statistical analysis is performed using Fold Change, exactTest using edgeR per comparison pair. The significant results are selected on conditions of |FC| ≧ 3 & exactTest *p*-value < 0.05.


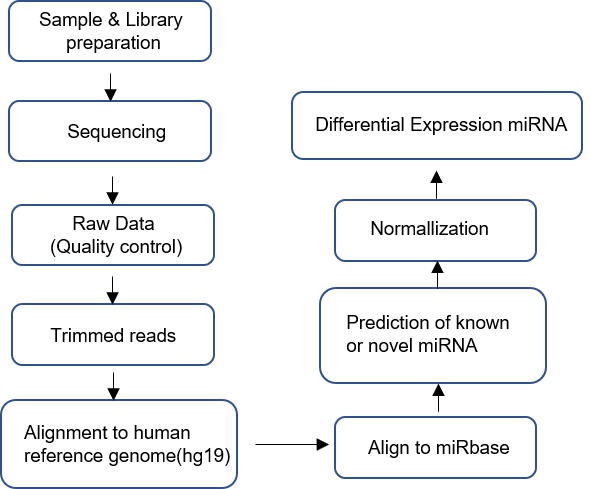


Next generation sequencing analysis pipeline.

**Appendix Tables**

**Table S1.** **The clinical information of patients with OSCC in CNUH cohort.**

| **Sex** | **Age** | **Main**  **subsite** | **T stage** | **N stage** | **M stage** | **ClinicalStage** | **Pathology** |
| --- | --- | --- | --- | --- | --- | --- | --- |
| M | 72 | tongue | 1 | 0 | 0 | I | Squamous cell carcinoma |
| F | 82 | tongue | 2 | 0 | 0 | II | Squamous cell carcinoma |
| M | 74 | buccal | 1 | 0 | 0 | I | Squamous cell carcinoma |
| M | 36 | tongue | 2 | 1 | 0 | III | Squamous cell carcinoma |
| M | 42 | tongue | 4a | 2b | 0 | IVa | Squamous cell carcinoma |
| M | 77 | tongue | 1 | 0 | 0 | I | Squamous cell carcinoma |
| M | 77 | tongue | 1 | 0 | 0 | I | Squamous cell carcinoma |
| M | 74 | tongue | 1 | 0 | 0 | I | Squamous cell carcinoma |
| M | 87 | RMT | 2 | 0 | 0 | II | Squamous cell carcinoma |
| M | 65 | RMT | 4a | 2c | 0 | IVa | Squamous cell carcinoma |
| M | 46 | tongue | 2 | 2b | 0 | III | Squamous cell carcinoma |
| M | 58 | tongue | 1 | 3 | 0 | IVa | Squamous cell carcinoma |
| F | 54 | tongue | 1 | 0 | 0 | I | Squamous cell carcinoma |
| F | 39 | tongue | 2 | 0 | 0 | II | Squamous cell carcinoma |
| F | 63 | tongue | 2 | 0 | 0 | II | Squamous cell carcinoma |
| M | 65 | tongue | 1 | 0 | 0 | I | Squamous cell carcinoma |
| M | 65 | tongue | 1 | 0 | 0 | I | Squamous cell carcinoma |
| M | 65 | tongue | 1 | 0 | 0 | I | Squamous cell carcinoma |
| M | 45 | tongue | 1 | 0 | 0 | I | Squamous cell carcinoma |
| F | 78 | tongue | 2 | 0 | 0 | II | Squamous cell carcinoma |
| F | 77 | tongue | 2 | 2b | 0 | IIIb | Squamous cell carcinoma |
| F | 69 | buccal,lip | 2 | 0 | 0 | II | Squamous cell carcinoma |
| M | 63 | FOM | 4a | 1 | 0 | IIIb | Squamous cell carcinoma |

M, male; F, female; RMT, retromolar trigone; FOM, floor of the mouth.

**Table S2.** **The clinical information of patients with OSCC in CNUH cohort for NGS.**

| **Sex** | **Age** | **Main**  **subsite** | **T stage** | **N stage** | **M stage** | **Clinical stage** | **Pathology** |
| --- | --- | --- | --- | --- | --- | --- | --- |
| F | 86 | Tongue | 2 | 0 | 0 | II | Squamous cell carcinoma |
| M | 66 | Buccal | 1 | 0 | 0 | I | Squamous cell carcinoma |
| M | 75 | Tongue | 3 | 2a | 0 | IVa | Squamous cell carcinoma |
| M | 54 | Tongue | 3 | 2a | 0 | IVa | Squamous cell carcinoma |

M, male; F, female.

**Table S3. The results of raw data statistics by next-generation sequencing**

| Sample ID | Total read bases | Total reads | GC (%) | Q20 (%) | Q30 (%) |
| --- | --- | --- | --- | --- | --- |
| Normal 1 | 2,633,386,224 | 51,635,024 | 36.19 | 91.94 | 85.52 |
| Normal 2 | 2,102,098,620 | 41,217,620 | 30.55 | 95.60 | 92.08 |
| Normal 3 | 2,235,461,478 | 43,832,578 | 29.36 | 95.88 | 92.63 |
| Normal 4 | 2,181,907,653 | 42,782,503 | 31.10 | 95.26 | 91.60 |
| Normal 5 | 2,206,288,458 | 43,260,558 | 32.33 | 87.31 | 77.28 |
| Normal 6 | 2,922,476,562 | 57,303,462 | 34.36 | 94.95 | 90.83 |
| Tumor 1 | 4,062,560,856 | 79,658,056 | 26.06 | 94.63 | 90.69 |
| Tumor 2 | 2,555,833,584 | 50,114,384 | 38.03 | 94.03 | 89.33 |
| Tumor 3 | 2,371,570,788 | 46,501,388 | 41.15 | 84.09 | 74.52 |
| Tumor 4 | 3,277,565,082 | 64,265,982 | 28.11 | 95.25 | 91.49 |

Total read bases: Total number of bases sequenced; Total reads: Total number of reads; GC (%): GC content; Q20 (%): Ratio of bases that have phred quality score greater than or equal to 20; Q30 (%): Ratio of bases that have phred quality score greater than or equal to 30.

**Table S4. microRNA-specific primers.**

|  |  | **ID** |
| --- | --- | --- |
| hsa-miR-92b-3p | miScript Primer Assay (Cat No.218300) | MS00032144 |
| hsa-miR-423-5p | miScript Primer Assay (Cat No.218300) | MS00009681 |
| hsa-miR-320c | miScript Primer Assay (Cat No.218300) | MS00041867 |
| hsa-miR-183-5p | miScript Primer Assay (Cat No.218300) | MS00031507 |
| hsa-miR-432-5p | miRCURY LNA miRNA PCR Assay (Cat NO. 339306) | YP00204776 |
| hsa-miR-92a-3p | miRCURY LNA miRNA PCR Assay (Cat NO. 339306) | YP00204258 |
| hsa-miR-629-5p | miRCURY LNA miRNA PCR Assay (Cat NO. 339306) | YP00204370 |
| hsa-miR-337-3p | miRCURY LNA miRNA PCR Assay (Cat NO. 339306) | YP00205938 |
| hsa-miR-199a-3p | miRCURY LNA miRNA PCR Assay (Cat NO. 339306) | YP00204536 |
| hsa-miR-199b-3p | 5′‐AACACGTGACAGTAGTCTGCA‐3′ |  |
| hsa-miR-16 | 5′‐GTAGCAGCACGTAAATATTGG‐3′ |  |

**Table S5. Differentially expressed miRNA by next-generation sequencing in serum from patients with OSCC and healthy controls**

| **Mature_Accession** | **Mature_ID** | **Fold Change** | **-log10(*p-*value)** | **Regulation** |
| --- | --- | --- | --- | --- |
| MIMAT0000092 | hsa-miR-92a-3p | 5.52007 | 5.968 | up |
| MIMAT0003218 | hsa-miR-92b-3p | 4.69249 | 4.299 | up |
| MIMAT0000261 | hsa-miR-183-5p | 4.67171 | 3.950 | up |
| MIMAT0004810 | hsa-miR-629-5p | 4.65223 | 2.272 | up |
| MIMAT0005793 | hsa-miR-320c | 4.43814 | 2.413 | up |
| MIMAT0000754 | hsa-miR-337-3p | -5.44630 | 1.795 | down |
| MIMAT0002814 | hsa-miR-432-5p | -5.11657 | 1.839 | down |
| MIMAT0000232 | hsa-miR-199a-3p | -3.37685 | 1.600 | down |
| MIMAT0004563 | hsa-miR-199b-3p | -3.37681 | 1.600 | down |

Up, indicates up-regulation; Down, indicates down-regulation.

**Appendix Figures and Figure Legends**

**Figure S1**

**
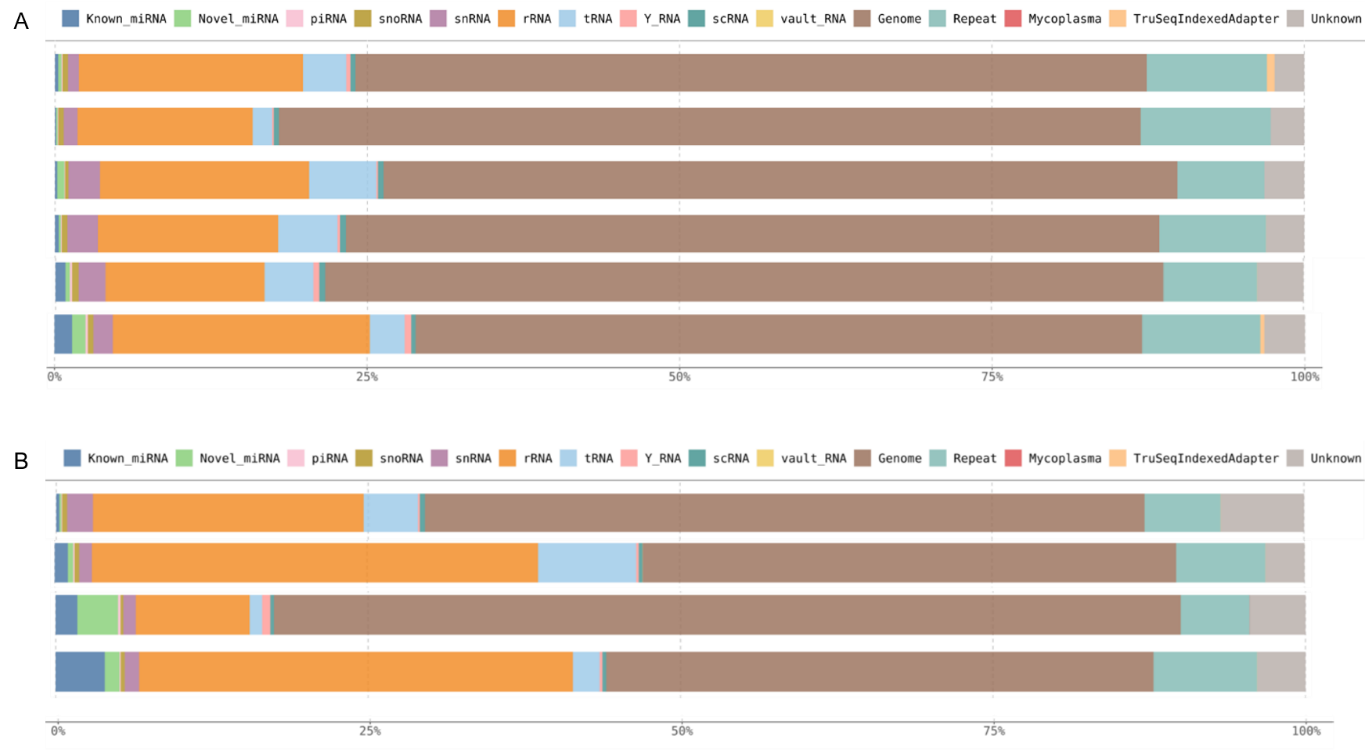
**

**Figure S1. Summary of small RNA composition of each sample.** The small RNA composition of (A) healthy control samples and (B) OSCC patient samples.

**Figure S2**


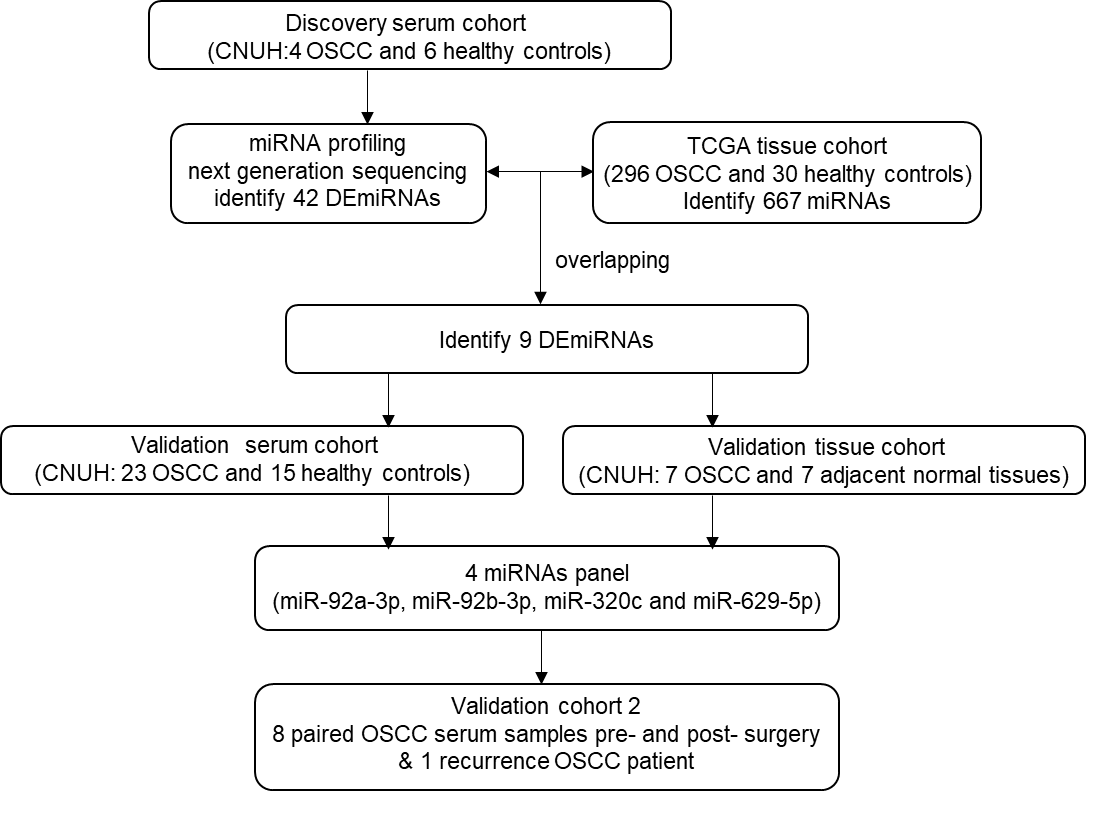


**Figure S2. An overview of the workflow of the research design.**

**Figure S3**


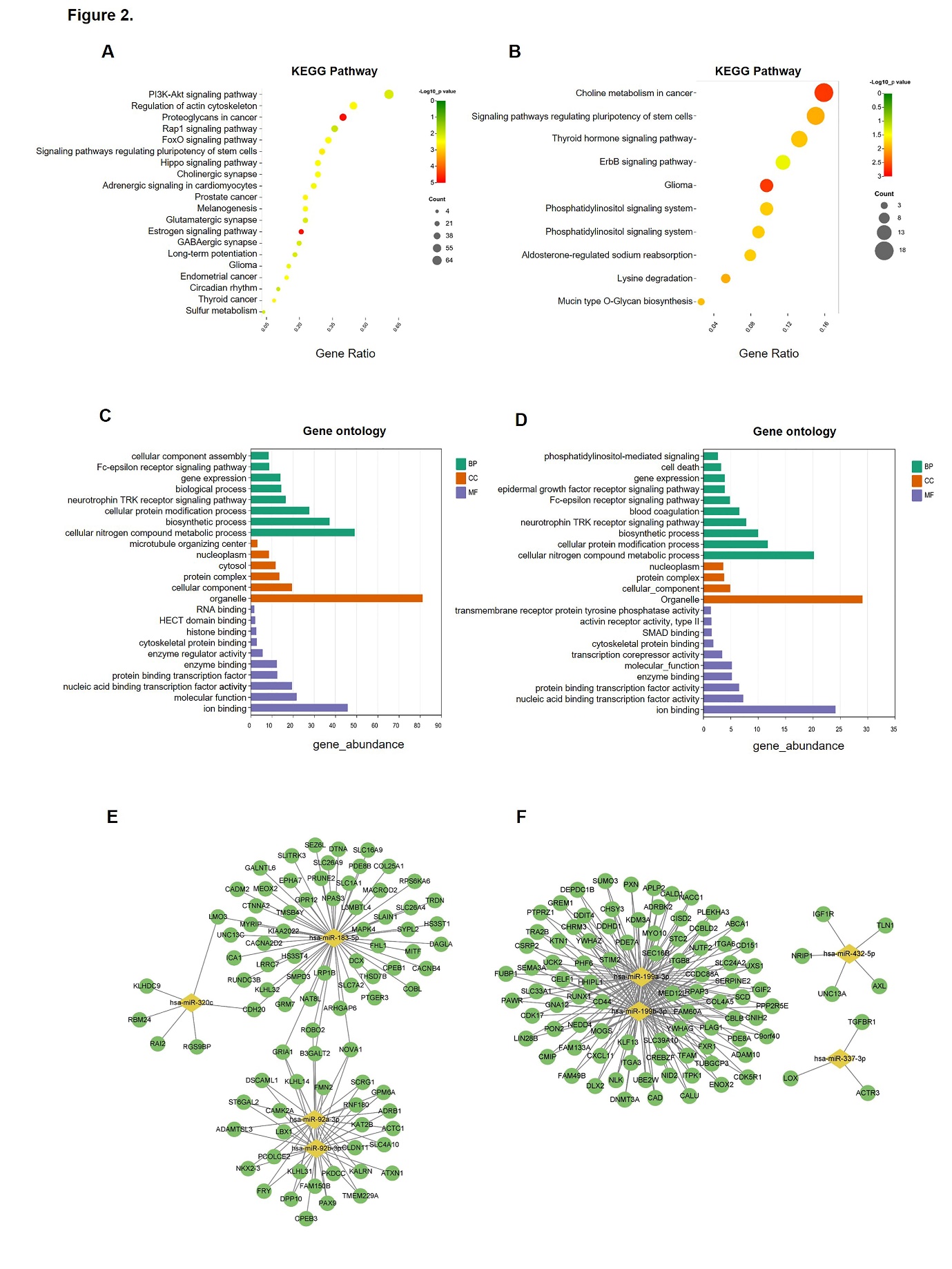


**Figure S3. Functional classification and linkage of the target genes of 9 DEmiRNAs.**

A-D: Kyoto Encyclopedia of Genes and Genomes (KEGG) pathway enrichment analysis and Gene Ontology (GO) functional annotation for the targeted genes of the 9 differentially expressed miRNAs (DEmiRNAs). The top 20 KEGG pathways affected by up-regulated miRNAs (A) and down-regulated miRNAs (B). The color of each dot denotes the *p*-value, and the size of each dot denotes the number of enrichment target genes. GO functional annotation including biological processes (BP), cellular components (CC), and molecular function (MF), which were affected by up-regulated miRNAs (C) and down-regulated miRNAs (D). The Y-axis indicates the enrichment score (p ≤ 0.05); E-F: miRNA-mRNA regulatory networks in OSCC. Up-regulated (E) and down-regulated (F) miRNA-mRNA network of the 9 differentially expressed miRNAs. Yellow diamonds represent miRNAs and green circles represent target mRNAs.

**Figure S4**


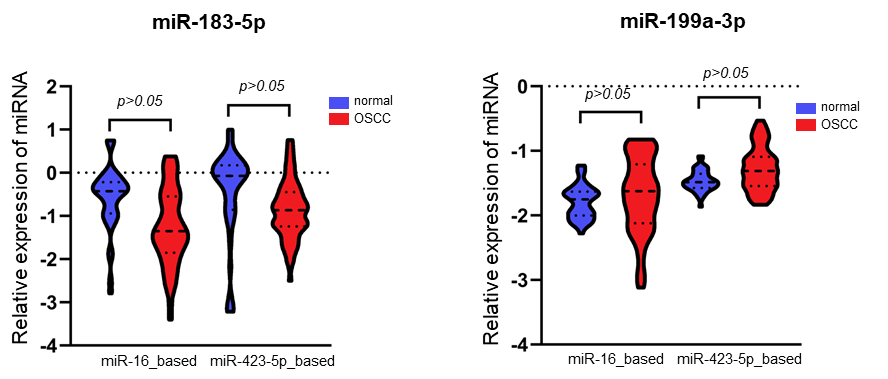


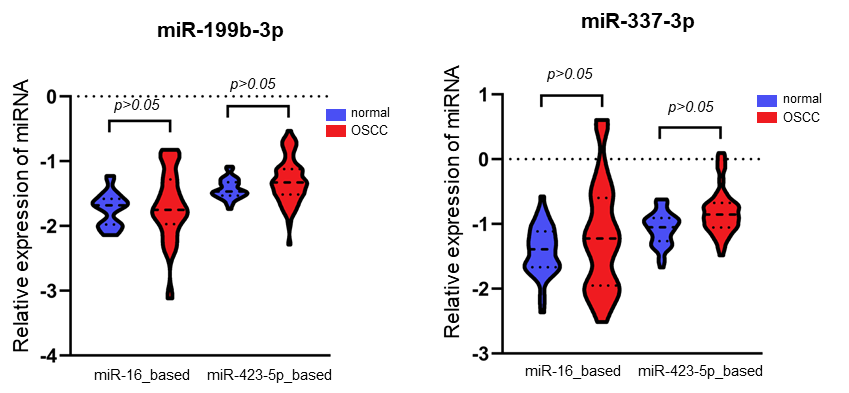


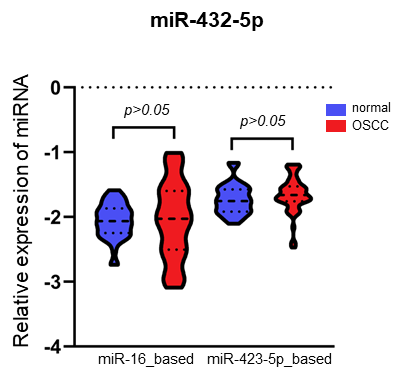


**Figure S4. Serum validation for selected DEmiRNAs.**

The expression levels of miRNAs were calculated using the 2^-ΔΔCT^ method, and the results are presented as relative log10 fold changes.

**Figure S5.**

**
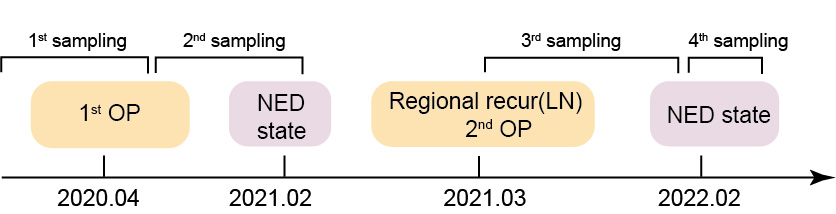
**

**Figure S5. The clinical course of recurrent patients with OSCC in CNUH cohort.**
